# Estimating and explaining cross-country variation in the effectiveness of non-pharmaceutical interventions during COVID-19

**DOI:** 10.1038/s41598-022-11362-x

**Published:** 2022-05-09

**Authors:** Nicolas Banholzer, Stefan Feuerriegel, Werner Vach

**Affiliations:** 1 ETH Zurich, Zurich, Switzerland; 2 LMU Munich, Munich, Germany; 3 Basel Academy for Quality and Research in Medicine, Basel, Switzerland; 4 University of Basel, Basel, Switzerland

**Keywords:** Public health, Epidemiology

## Abstract

To control the COVID-19 pandemic, countries around the world have implemented non-pharmaceutical interventions (NPIs), such as school closures or stay-at-home orders. Previous work has estimated the effectiveness of NPIs, yet without examining variation in NPI effectiveness across countries. Based on data from the first epidemic wave of $n=40$ countries, we estimate country-specific differences in the effectiveness of NPIs via a semi-mechanistic Bayesian hierarchical model. Our estimates reveal substantial variation between countries, indicating that NPIs have been more effective in some countries (e. g. Switzerland, New Zealand, and Iceland) as compared to others (e. g. Singapore, South Africa, and France). We then explain differences in the effectiveness of NPIs through 12 country characteristics (e. g. population age, urbanization, employment, etc.). A positive association with country-specific effectiveness of NPIs was found for government effectiveness, gross domestic product (GDP) per capita, population ages 65+, and health expenditures. Conversely, a negative association with effectiveness of NPIs was found for the share of informal employment, average household size and population density. Overall, the wealth and demographic structure of a country can explain variation in the effectiveness of NPIs.

## Introduction

Countries around the world have resorted to non-pharmaceutical interventions (NPIs) in order to control transmission of SARS-CoV-2. As in Brauner et al.^1^, we refer to NPIs in this study as population-level public health interventions taken by governments to reduce the number of person-to-person contacts. Commonly implemented NPIs in this regard are the closure of schools, venues and workplaces, as well as gathering bans and stay-at-home orders^1–3^.

Previous studies have estimated the effectiveness of NPIs during the first epidemic wave^4^. Thereby, many studies focused on individual countries and assessed the effectiveness of the specific measures taken by their governments^5–11^. Some studies also estimated the effects of a comparable set of NPIs across multiple countries^1,3,12,13^. These studies exploit both within and between country-variation in the implementation of NPIs over time. However, they provide only pooled estimates and do not examine country-specific variation in the effectiveness of NPIs.

Differences in the effectiveness of NPIs between countries can help in identifying conditions that may be favorable or unfavorable for NPIs to control transmission. This could impact public health decision making in to ways. First, governments may be able to change some conditions in order to improve preparedness for future pandemics (e. g. by increasing health expenditures), which could also imply a more favorable environment for the effectiveness of NPIs. Second, for conditions that can or will not change in the short term (e. g. the demographic structure of the population), a better understanding of how NPIs vary in their effectiveness across countries is still relevant for governments that want to introduce an NPI in one country based on findings that have quantified the effectiveness of that NPI in another country. When transferring results, public health decision makers may thus need to assume a higher (or lower) effectiveness of NPIs for their respective country.

To identify (un-)favorable conditions for the effectiveness of NPIs, we can attribute differences in the effects of NPIs between countries to certain characteristics of the countries (e. g. income, population age, and household size). Such characteristics have been used previously to explore variation in the severity of the COVID-19 pandemic across countries^14–17^, but not for comparing the effectiveness of NPIs across countries. Analyzing the latter is motivated by findings that the individual socioeconomic status can determine the risk of infection and can also influence the effectiveness of NPIs^18^.

In this study, we hypothesize that there is variation in the effectiveness of NPIs across countries, and that this variation can be explained by country characteristics. Specifically, our hypothesis is that NPIs have overall been more effective in some countries as compared to others, and that differences in the effects of NPIs are associated with country characteristics. For example, we expect that NPIs have been more effective in rich countries where people may find it easier to stay and work from home. Contrary to this, we expect that NPIs have been less effective in more densely populated countries where it is generally more difficult to reduce the number of person-to-person contacts. We provide a detailed hypotheses development in the "Hypotheses development" Section.

**Figure 1:**
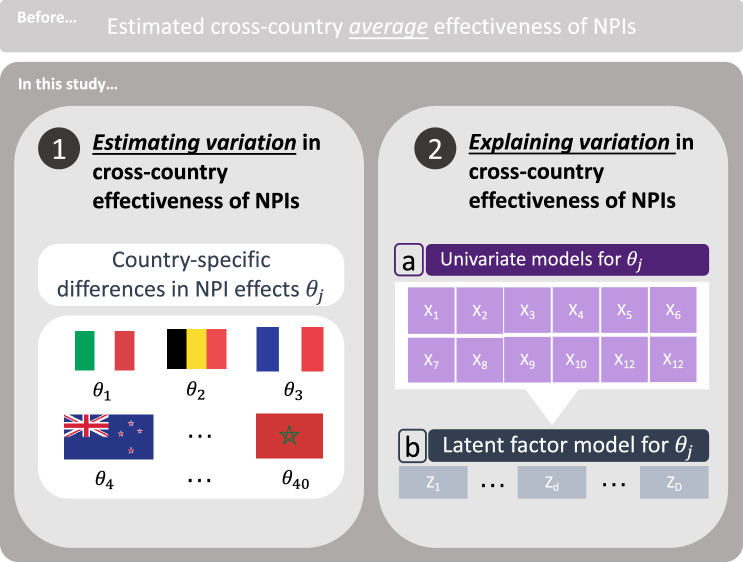
Visual summary of the two-step analysis for estimating and explaining variation in cross-country effectiveness of NPIs. Step $\textcircled {1}$: Estimating variation in cross-country effects of NPIs $\theta _1, \dots , \theta _{40}$. Step $\textcircled {2}$: Explaining variation in $\theta _j$ through 12 country characteristics $X_1,\dots ,X_{12}$. Association between characteristics and $\theta _j$ is estimated as follows: $\boxed {\mathbf{a}}$ by linking $\theta _j$ separately to each country characteristic, and $\boxed {\mathbf{b}}$ by linking $\theta _j$ to latent factors $Z_1, \dots , Z_D$, which are determined with a latent factor model.

We approach our hypotheses in two steps (Fig. 1). In Step $\textcircled {1}$, we estimate differences in the effectiveness of NPIs between countries by extending an established semi-mechanistic Bayesian hierarchical model that was used for estimating the cross-country average effects of NPIs^3^. The model is fitted to data from Brauner et al.^1^, which is publicly available, high-quality data on systematically coded interventions for a large number of $n=40$ countries covering the first epidemic wave of COVID-19. In Step $\textcircled {2}$, we estimate the association between the country-specific NPI effect and a set of 12 different country characteristics (e. g. population age, urbanization, employment, etc.) inside our Bayesian hierarchical model. This allows us to identify characteristics of countries where NPIs have been more or less effective. For better interpretability, we eventually map the different country characteristics onto a lower-dimensional representation via a latent factor model, which identifies two overarching factors of relevance, namely the wealth and demographic structure of a country.

## Materials and methods

### Data

Reported SARS-CoV-2 cases for each country between February and May 2020 were obtained from the Johns Hopkins Coronavirus Resource Center^19^. Data on the implementation dates of NPIs are obtained from Brauner et al.^1^. Therein, the authors collected and systematically coded non-pharmaceutical interventions for a large sample of $n=40$ countries. We used the same data due to the fact that the work by Brauner et al. was highly influential for subsequent analyses. In particular, since the data has already been used by Brauner et al. for extensive study of the average effects of NPIs, we can build on their work and focus on studying the differences in the effects of NPIs between countries.

One small country (Andorra) was excluded from the analysis since information on most characteristics was not available. The 8 considered NPIs are school and university closures, gathering bans of three different sizes, the closure of some or most high-risk face-to-face businesses, and stay-at-home orders. Country characteristics were collected from publicly available sources (Table 1). We considered a set of 12 characteristics that could be associated with the effectiveness of NPIs. The rationale for the selection is discussed in the following.

**Table 1:** List of country characteristics.

Variable	Definition	Source (year)
GDP per capita	Gross domestic product (USD) per capita	World Bank (2019)
Population ages 0–14	Share of population ages 0–14 (% of total population)	World Bank (2019)
Population ages 15–64	Share of population ages 15–64 (% of total population)	World Bank (2019)
Population ages 65+	Share of population ages 15–64 (% of total population)	World Bank (2019)
Health expenditures	General government health expenditures (% of GDP)	World Bank (2018)
Urban population	Population living in urban area (% of total population)	World Bank (2019)
Population density (log)	Log number of people per square km	World Bank (2018)
Government effectiveness	Government Effectiveness Indicator	World Bank (2019)
Employment in services	Employment in accommodation and food service activities (% of total employment)	International Labour Organization (2019)
Informal employment	Employment outside the formal sector (% of total employment)	International Labour Organization (2018)
Average household size	Average household size	Population Reference Bureau (2020)
GHS score	Overall score in “Global Health Security Index” assessing global health security capabilities	Johns Hopkins Center for Health Security, the Nuclear Threat Initiative and the Economist Intelligence Unit (2019)

GDP: gross domestic product; GHS: global health security.

### Hypotheses development

Our selection of country characteristics was largely inspired by investigations into the association between country characteristics and the severity of the pandemic^14–17^. Below, we discuss the hypotheses regarding how our selected country characteristics may be associated with the effectiveness of NPIs (Table 2).

The country characteristics we expected to be positively associated with the effectiveness of NPIs include gross domestic product (GDP) per capita, population ages 65+, health expenditures, government effectiveness and global health security (GHS) score. It is reasonable to expect that the effectiveness of government interventions should correlate with general indicators of government effectiveness. Moreover, better preparedness for a pandemic as expressed by a high score on the global health security index and high governmental health expenditures can imply that NPIs are more effective in such countries. GDP per capita was selected as a proxy for income, which has been shown to be positively associated with the effectiveness of lockdowns in Chilean municipalities^20^. A higher share of the elderly population may increase the effectiveness of NPIs as previous studies indicate greater compliance with social distancing measures among older age groups^21–23^. More employment in services may be associated with greater effectiveness of NPIs concerning business and venue closures. The reason is that points-of-interest such as bars and restaurants exhibit greater risk of infection^24^, so that business and venue closures may be relatively more effective in such workplaces as compared to workplaces that require fewer face-to-face interactions.

The country characteristics we expected to be negatively associated with the effectiveness of NPIs include population ages 0–14, population ages 15–64, urban population, population density, employment in services, informal employment, and average household size. NPIs may be less effective in countries with a younger population that is less compliant with social distancing measures than the older population. Furthermore, a higher share of the working age population may decrease the effectiveness of NPIs. In this regard, previous work showed that the working age group contributed disproportionally to transmission in the US^25^, probably because working adults had to continue working to support their families. This effect may be amplified by informal employment, where working adults cannot rely on social benefits to compensate income losses. Finally, population density, urban population and average household size are indicators of the living environment. Previous work showed that population density is positively associated with the reproduction number^26,27^, with findings suggesting that more stringent policy measures may be needed to offset the effects of large population densities^27^. The association is probably similar for urban populations as a larger urban population often goes hand in hand with larger population densities. Larger households could impede the effectiveness of NPIs, especially because NPIs are not targeting within-household transmission. Previous work showed that larger households were associated with more secondary cases^28^ and that the proportion of positive COVID-19 test results in Leicester (UK) was higher among larger households after the lockdown^29^.

**Table 2:** Hypotheses regarding the association between country-specific NPI effects and country characteristic. Positive (negative): Increase in variable increases (decreases) country-specific NPI effect.

Variable	Expected association
GDP per capita	Positive
Population ages 0–14	Negative
Population ages 15–64	Negative
Population ages 65+	Positive
Health expenditures	Positive
Urban population	Negative
Population density (log)	Negative
Government effectiveness	Positive
Employment in services	Positive
Informal employment	Negative
Average household size	Negative
GHS score	Positive

GDP: gross domestic product; GHS: global health security.

### Model overview

Our model builds on the semi-mechanistic Bayesian hierarchical model used by Banholzer et al.^3^ to estimate the cross-country average effects of NPIs. In the following, we summarize the specification of this model ("Model overview" Section) and then describe how we adapted it for our two steps: $\textcircled {1}$ estimating variation in the effects of NPIs across countries ("[Sec Sec6]" Section); and $\textcircled {2}$ explaining this variation through country characteristics ("[Sec Sec7])" Section).

**Figure 2:**
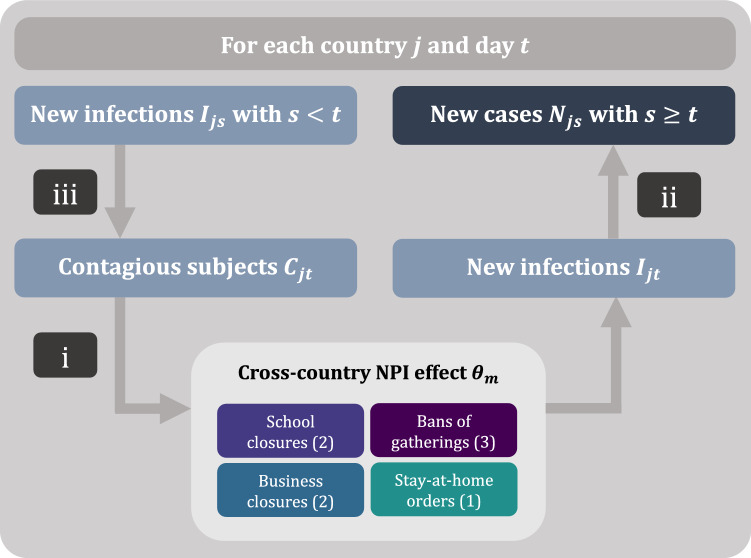
Visual summary of the model structure. The three main components are: (i) The number of new infections is modelled as a function of the number of contagious subjects, the country-specific daily transmission rate, and the reductions from 8 active NPIs (school and university closures, bans of gatherings larger than 10, 100, and 1000 people, closure of some or most high-risk face-to-face businesses, and stay-at-home orders). (ii) The observed number of new cases is a weighted sum of the number of new infections in the previous days. (iii) The number of contagious subjects is a weighted sum of the number of new infections in the previous days.

Figure 2 provides a visual summary of our model structure. Our model links two unobserved quantities (i. e. the daily number of contagious subjects and the daily number of new infections) to an observed quantity (i. e. the number of reported new cases). The links consist of three components: (i) a regression type model relating the number of new infections to the number of contagious subjects, the country-specific daily transmission rate, and the presence of active measures; (ii) a link between the number of new infections to the number of reported new cases; and (iii) a link between the number of new infections and the number of contagious subjects. We detail the three components (i)–(iii) in the following.

(i) In the first component, the fundamental part is a model for the expected number $\mu$ of new infections $I_{jt}$ in country *j* at day *t*. In the absence of any measure, this would be $\mu ^{I_{jt}} = C_{jt} \ \exp {\left( \alpha + \alpha _j\right) }$, where $C_{jt}$ are the number of contagious subjects and $\alpha + \alpha _j$ is the country-specific daily transmission rate. The presence of NPIs leads to reductions from avoided infections and $\mu ^{I_{jt}}$ is given by

1 $$\begin{aligned} \mu ^{I_{jt}} = C_{jt} \cdot \exp {\left( \alpha + \alpha _j + \sum _{m=1}^{M} \theta _m \, D_{mjt}\right) }, \end{aligned}$$

where $\theta _{m}$ is the cross-country average effect of measure $m = 1, \dots , M$ and $D_{mjt}$ is a dummy variable that equals one if measure *m* is implemented in country *j* at time *t*.

(ii) In the second component, the expected number of new cases is calculated as a weighted sum of the number of new infections in the previous days. The weights reflect the distribution of the time from infection to reporting. This distribution is estimated from the data incorporating prior knowledge about the incubation period and the reporting delay. The observed number of new cases is then modeled as a negative binomial distribution with the specified mean, allowing for overdispersion.

(iii) In the third component, the number of contagious subjects is calculated as a weighted sum of the number of new infections in the previous days. The weights reflect the probability of being contagious on a specific day after being infected and can be determined from the generation time distribution. This distribution is assumed to be known and our choice is based on an estimate by a study using data on the exposure for both the index and secondary case^30^.

### Modeling variation in the effects of NPIs across countries (Step $\textcircled {1}$)

In Step $\textcircled {1}$ of this study, we want to estimate the country-specific effects of NPIs $\theta _{mj}$. However, each country implemented a specific NPI during the first wave only once, and, hence, we end up having only one single exposure carrying all information to estimate each of the parameters $\theta _{mj}$. In addition, NPIs were often implemented in close succession or even on the same day within countries. In order to model country-specific effects of NPIs, we thus make a simplification by dividing $\theta _{mj}$ into a NPI-specific and a country-specific effect

2 $$\begin{aligned} \theta _{mj} = \theta _m + \theta _j, \end{aligned}$$

where $\theta _m$ is the average cross-country NPI effect of measure *m* and $\theta _j$ is the country-specific NPI effect of country *j*. That is, the country-specific effect is the same for all NPIs *m* in country *j*. In other words, we consider the case where NPIs were overall more or less effective in country *j*. The country-specific NPI effect is modelled as the deviation from the average cross-country NPI effect, i. e. $\theta _j \sim \mathrm {Normal}(0, \tau )$, where $\mathrm {E}(\theta _j)=0$ and $\tau$ quantifies the variation in the effectiveness of NPIs between countries.

### Modeling country-specific NPI effects as a function of country characteristics (Step $\textcircled {2}$)

In Step $\textcircled {2}$ of this study, we want to estimate the association between country-specific NPI effects and country characteristics. It is difficult to estimate the effects of all country characteristics in one multiple regression model because the variables exhibit strong correlations. Therefore, we conduct two analyses. In analysis $\boxed {\mathbf{a}}$, we estimate the univariate effect of country characteristics on the country-specific NPI effect. That is, the model is estimated separately for each variable. In analysis $\boxed {\mathbf{b}}$, we specify a latent factor model, which allows us to jointly estimate the association between country characteristics and the country-specific NPI effect.

$\boxed {\mathbf{a}}$ **Univariate associations between country-specific NPI effects and characteristics**

To estimate the univariate association between country-specific NPI effects and country characteristics, we link the country-specific effect $\theta _j$ separately to one of the country characteristic $X_k$, i. e.

3 $$\begin{aligned} \theta _j = \beta _k X_{jk} + \epsilon _j \quad k = 1, \dots , K, \end{aligned}$$

where each $\beta _k$ estimates the association between the country-specific NPI effect and the value $X_{jk}$ of the centered variable with the country characteristic $X_1, \dots , X_K$, and $\epsilon _j$ is the residual country-specific effectiveness *un*-explained by $X_{jk}$.

A first inspection of the country-specific NPI effects $\theta _j$ indicated that they are correlated with the country-specific intercept $\alpha _j$. Since $\alpha + \alpha _j$ reflects the country-specific transmission rate in the absence of NPIs in country *j*, we have to expect that many of the country characteristics show an association with this rate. Consequently, we decided to model $\theta _j$ explicitly as a function of $\alpha _j$, i. e.

4 $$\begin{aligned} \theta _j = \psi \alpha _j + \beta _k X_{jk} + \epsilon _j \quad k = 1, \dots , K, \end{aligned}$$

where $\psi$ estimates the association between $\theta _j$ and $\alpha _j$. Modeling $\theta _j$ as a function of $\alpha _j$ ensures that $\beta _k$ describes the association of $X_k$ with the effectiveness of the NPIs, and not with the transmission rate.

$\boxed {\mathbf{b}}$ **Associations between country-specific NPI effects and latent factors**

To estimate the joint effect of country characteristics *X* on the country-specific NPI effect $\theta _j$, we specify a latent factor model. An alternative name for the latent factor model is Bayesian principal component analysis^31^. The idea of our latent factor model is to represent the observed country characteristics *X* in a lower-dimensional space by latent factors *Z*, which are then linked to the country-specific NPI effects (i. e. *Z* replaces *X* in Eq. 4). Formally, the latent factor model uses a linear transformation to represent the observed data $X_1, \dots , X_K$ by independent and identically distributed latent factors $Z_1, \dots , Z_D \sim \mathrm {Normal}(0, 1)$, i. e.

5 $$\begin{aligned} X_{jk} = \sum _{d=1}^D W_{kd} \, Z_{jd} + \epsilon _k, \end{aligned}$$

where $W_{kd}$ is the weight of the *k*-th country characteristic on latent factor $d =, 1 \dots , D$, and $\epsilon _k$ is the residual variation. The matrix *W* is lower triangular and $C=WW^T + \sigma ^2 I_k$ is the covariance matrix of the observed country characteristics *X*. The number of latent factors *D* can be determined automatically by introducing a parameter $\omega _d$ controlling the variation of each column vector $W_d$, such that for small $\omega _d$ the weights $W_d$ are effectively zero (i. e. removing dimension *d*).

Note that, for both the univariate analysis and the latent factor model, all variables with the country characteristics were standardized by their empirical mean and standard deviation in order to have their effects on a comparable scale. There was no information on informal employment for five countries. The missing values for this variable were modeled with a standard normal distribution as prior.

Of note, we aim to explain variation in the effectiveness of NPIs by estimating the association between country-specific NPI effects and country characteristics. As such, our estimations should be interpreted as being associative (rather than causal) links.

### Parameter estimation

Detailed prior and modeling choices are provided in SI Appendix A. All model parameters were estimated with a semi-mechanistic Bayesian hierarchical model. Specifically, Markov chain Monte Carlo (MCMC) sampling is used as implemented by the Hamiltonian Monte Carlo algorithm with the No-U-Turn sampler (NUTS) from the probabilistic programming language Stan, version 2.21.0^32^. Each model was estimated with 4 Markov chains and 2,000 iterations of which the first 1,000 iterations were discarded as part of the warm-up. Estimation power was evaluated via the effective sample size ${\hat{n}}_{\text {eff}}$, and convergence of the Markov chains was assessed with the Gelman-Rubin convergence diagnostic (${\hat{R}}$). If not stated otherwise, we report posterior means and credible intervals (CrIs) based on the 2.5% and 97.5% quantile of the posterior samples.

The result section presents estimation results for the country-specific NPI effects $\theta _j$ and their association with country characteristics. The cross-country average effects of NPIs are not shown as they have already been extensively studied elsewhere^1,3^.

### Simulation-based study

Simulation-based studies in previous work demonstrated that semi-mechanistic Bayesian hierarchical models can recover the true cross-country NPI effects $\theta _m$^1,3^. Analogously, a simulation-based study was performed in this work to demonstrate that our model can recover the true country-specific effects $\theta _j$ within the uncertainty implied by the fitted posterior distribution of our model. The results indicate that our model recovers the true country-specific effects (SI Appendix B). We observe that some $\theta _j$ are pulled towards zero, especially for countries with smaller numbers of cumulative cases. This is the consequence of shrinkage, which is inherit by our model as the $\theta _j \sim \mathrm {Normal}(0, \tau )$ are modeled as varying slopes with a weakly informative prior on the hyperparameter $\tau$. Nevertheless, there is a tendency for the model to shrink positive $\theta _j$ more towards zero than negative $\theta _j$. This asymmetry could be the result of our asymmetric prior for $\theta _m$ (SI Appendix A).

## Results

### Estimated country-specific effectiveness of NPIs (Step $\textcircled {1}$)

This study consists of two steps (Fig. 1). Step $\textcircled {1}$ is to estimate country-specific variation in the effectiveness of NPIs using a semi-mechanistic Bayesian hierarchical model (see Materials and methods). This should inform whether there were some countries where the effects of NPIs were more effective and some countries where they were less effective than the cross-country average.

**Figure 3:**
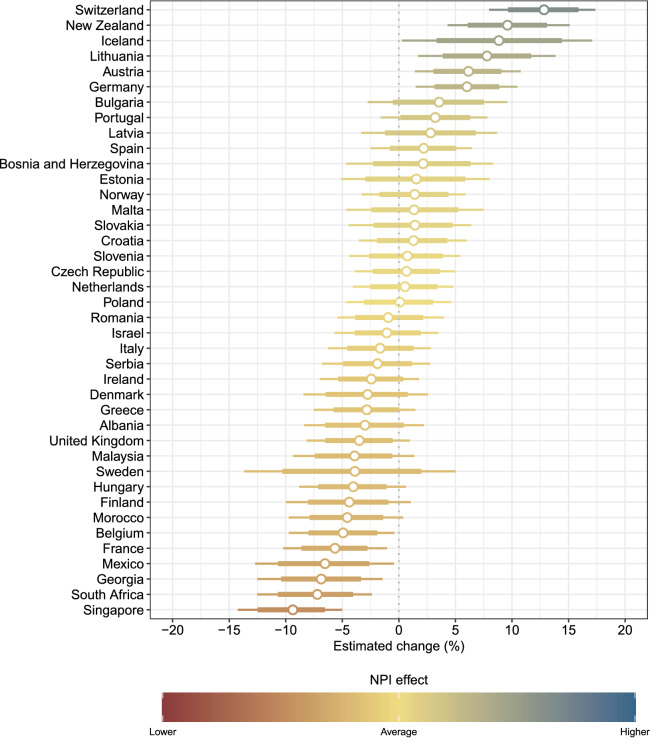
Estimated relative change (in %) in avoided new infections compared to the cross-country average effect of the single NPIs (posterior mean as dots with 80% and 95% credible interval as thick and thin lines, respectively).

The country-specific NPI effect is quantified as the relative change (in %) in avoided new infections compared to the cross-country average effect of the single NPIs. A positive change indicates that NPIs have overall been more effective in this country than the cross-country average. Conversely, a negative change indicates that NPIs have overall been less effective in this country than the cross-country average. The estimated country-specific NPI effects are shown in Fig. 3. For some countries, the NPIs have overall been more effective as compared to the cross-country average. Examples are Switzerland, New Zealand and Iceland. For some other countries, the NPIs have overall been less effective as compared to the cross-country average. Examples are Singapore, South Africa, and France.

Incorporating country-specific NPI effects also leads to a clear improvement in the model fit. Model comparison according to the leave-one-out cross-validated expected log predictive density (a measure for the goodness-of-fit of the predictive distribution) using Pareto-smoothed importance sampling^33^ indicates a strong improvement in model fit compared to the model without country-specific NPI effects (difference = 39.5, standard error = 6.8). The parameter estimating the variation in country-specific NPI effects is also distinguishable from zero (posterior standard deviation = 0.052, 95% CrI: 0.038 to 0.071), indicating significant heterogeneity in the effectiveness of NPIs across countries.

### Explaining the effectiveness of NPIs via country characteristics (Step $\textcircled {2}$)

Step $\textcircled {2}$ of this study is to explain the variation in the effectiveness of NPIs between countries using country characteristics. For this, we make use of 12 country characteristics (e. g. population age, urbanization, employment, etc.; see Materials and methods). Subsequently, we extend the analysis using a latent factor model to find a lower-dimensional representation among the country characteristics that allows for better interpretability.

$\boxed {\mathbf{a}}$ **Univariate associations between country-specific NPI effects and country characteristics**

**Figure 4:**
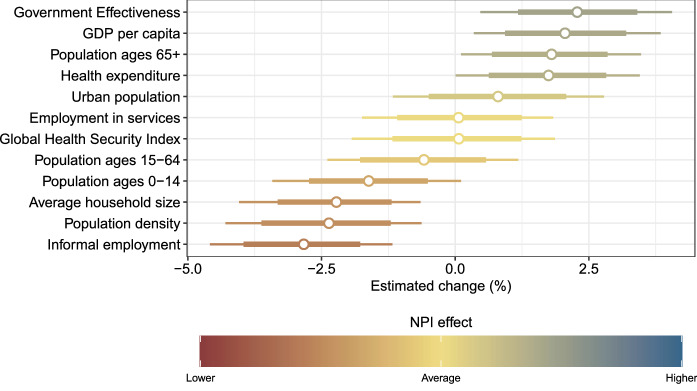
Estimated relative change (in %) in avoided new infections compared to the cross-country average effect of the single NPIs (posterior distribution with mean as dots and with 80% and 95% credible interval as thick and thin lines, respectively) for a $+$1 standard deviation (SD) increase in the variable with the country characteristic.

The association between country-specific NPI effects and country characteristics is similarly quantified as the relative change (in %) in avoided new infections compared to the cross-country average effect of the single NPIs for a $+$1 standard deviation (SD) increase in the variable with the country characteristic. The estimated univariate associations between the effectiveness of NPIs and country characteristics are shown in Fig. 4.

Government effectiveness (2.3%, 95% CrI: 0.5% to 4.1%), GDP per capita (2.1%, 95% CrI: 0.3% to 3.8%), population ages 65+ (1.8%, 95% CrI: 0.1% to 3.5%), and health expenditures (1.7%, 95% CrI: 0.0% to 3.5%) were positively associated with the country-specific NPI effect, indicating that larger values for these country characteristics were associated with higher effectiveness of NPIs. In contrast to that, informal employment (−2.8, 95% CrI: −1.2 to −4.6), population density (−2.4%, 95% CrI: −0.6% to −4.3), and average household size (−2.2, 95% CrI: −0.6 to −4.0) were negatively associated with the country-specific NPI effect, indicating that larger values for these country characteristics were associated with lower effectiveness of NPIs.

$\boxed {\mathbf{b}}$ **Associations between country-specific NPI effects and latent factors**

**Figure 5:**
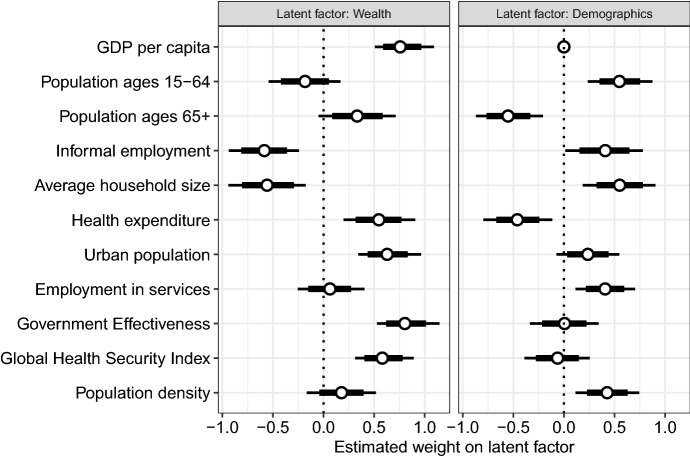
Estimated weight on latent factors (posterior mean as dots with 80% and 95% credible interval as thick and thin lines, respectively).

Many of the country characteristics exhibit strong correlations (SI Fig. S3). Thus, one would expect to obtain similar estimates for their associations with country-specific NPI effects. To condense the information shared by multiple country characteristics, a latent factor model was estimated. Automatic determination of the latent dimensional space resulted in two latent factors. The estimated weights on these latent factors are shown in Fig. 5.

The first latent factor may be interpreted as the wealth of a country (i. e. high GDP per capita, high health expenditures, high health security, high government effectiveness and low informal employment), and the second latent factor characterizes a specific demographic structure (i. e. young, dense, urban populations living in larger households and working more informally). We thus refer to these factors as “wealth” and “demographics” in the following. Wealth was associated with a positive change in the country-specific NPI effect (1.9%, 95% CrI: −0.6% to 4.4%), while the specific demographic structure was associated with a negative change in country-specific NPI effect (−1.7%, 95% CrI: 0.9% to −4.5%). However, some uncertainty about the direction of the effects has to be acknowledged, as both 95% credible intervals include 0%.

The latent factor model allows to quantify the improvement in the proportion of variance that is explained by country characteristics. For this, the percentage change in the variation of the country-specific NPI effects between the model with and without the latent factors is computed. There is a strong reduction in the variation (−65% 95% CrI: −7% to −94%), which suggests that country characteristics explain a significant proportion of the variation in the country-specific NPI effects.

Our estimated associations are based on observations from $n=40$ countries, and only some countries have an estimate for the country-specific NPI effect that is distinguishable from zero (Fig. 3). One may thus be concerned that our estimated associations are largely driven by single countries. To investigate this, a leave-one-country-out sensitivity analysis was conducted for the latent factor analysis (SI Appendix D). Thereby, the two latent factors from the model using all countries were fixed at their posterior mean and used as the country characteristics. The association between the country-specific NPI effects and these latent factors was then subject to the leave-one-country-out analysis where the model was re-estimated leaving out one country at a time. The results for the effects of the first latent factor (wealth) are slightly sensitive to leaving out Switzerland while the second latent factor (demographics) is slightly sensitive to leaving out Singapore (SI Fig. S4). All other countries had only a minor influence on the results.

## Discussion

### Interpretation

Our estimates suggest that there were some countries where NPIs were relatively more effective and some countries where they were less effective than the cross-country average. Of note, we compare the effectiveness of NPIs – and not the overall effectiveness of countries in managing the pandemic. The latter is related to how many and when NPIs were implemented. In contrast, our research is motivated by the need for a better understanding of whether NPIs might be more effective in countries due to their government, health structure, or other socioeconomic variables. For example, our results suggest that NPIs have been highly effective in Switzerland, yet NPIs were implemented comparably late into the pandemic, thus explaining why Switzerland nevertheless has a high number of cumulative cases. In general, there is (or can be) a difference between the effectiveness of NPIs and the severity of a country’s pandemic. Our work attempts to estimate country-specific NPI effects and their associations with country characteristics. This is different from work estimating the association between country characteristics and the severity of the pandemic^14–17^.

The second step of this study aimed at identifying characteristics that explain the variation in the effectiveness of NPIs between countries. Positive associations were found for government effectiveness, GPD per capita, population ages 65+, and health expenditures. These align with our hypotheses based on related work (see "Hypotheses development" Section in Materials and methods) that NPIs may be more effective when governments are more effective or spend more on health care, or when countries have higher income^20^ or a higher share of the older population that may be more compliant with the measures^21–23^.

Negative associations were found for population density, informal employment, and average household size. These also align with our hypotheses based on related work that NPIs may be less effective in more densely populated areas^26,27^, in countries with larger households^28,29^, and in countries where a larger share of informally employed workers who may continue working to prevent income losses at the risk of contracting and transmitting the disease^25^.

We found no effect for other country characteristics that was distinguishable from zero, despite theoretical arguments. Similar to population density, we would have expected a negative association between the effectiveness of NPIs and the share of the urban population. Employment in services was considered following our expectation that NPIs concerning business and venue closures may have greater impact in countries with a higher share of the population employed in accommodation and food service activities. Yet, we acknowledge that this variable may not be the best proxy for the number of people getting together at points-of-interest that exhibit greater risk of infection^24^. Finally, the global health security index (or similar indices) should indicate how well a government is prepared or able to deal with a pandemic and thus a positive association was expected. Yet, concerns were already raised before this study about the ranking of some countries in the index, in particular the United Kingdom and the United States were ranked at the top of the index but responded much worse to the pandemic than other countries^34^. Nevertheless, the fact that some associations are counterintuitve should stress the point that all associations should be treated with caution and not considered as causal links to the effectiveness of NPIs.

Results from our latent factor analysis showed that our country characteristics could be summarized by two latent factors, namely wealth and demographics. Higher wealth was positively associated with the effectiveness of NPIs, probably because wealthier countries have more measures and funding to deploy that ensure higher compliance with NPIs (e. g. financial compensation for workers staying home). Relatedly, it was found that mobility reductions were more pronounced in wealthier areas^35^ and that socioeconomic inequalities correlated with different growth in incidence following social distancing^36^. The demographic structure – in the form of a younger population, living in more densely populated and urban areas, as well as in greater households – was negatively associated with NPI effectiveness. A probable reason is that social distancing in such settings is comparatively more difficult. For example, overcrowded spaces (e. g. dense areas or large households) facilitate transmission of infectious respiratory diseases^37,38^ and thus limit the effectiveness of NPIs.

### Limitations

Our study has several limitations. First, the general modeling approach chosen may overestimate the overall effect of the NPIs^3^. This is because any change in the transmission rate is explained by the effect of the NPIs, although also other factors may have contributed to this. On the other side, there is a risk of underestimation, as the model assumes a change just at the time when NPIs are implemented. For example, studies have shown that changes in mobility^39,40^ or transmission rates^40–42^ oftentimes occurred before the implementation of NPIs. In general, these deficiencies of the general model may – to some degree – also explain country-specific effects if certain model violations are more pronounced in individual countries. Along these lines, our results should be interpreted as associative and not as causal estimates.

Moreover, our model only incorporates an overall country-specific effect. That is, the country-specific effect is not varying by NPI. It would be valuable to estimate country-specific effects for each NPI as some country characteristics may explain more variation between countries for some particular NPIs (e. g. informal employment and employment in services for business closures). However, our data provides limited information to estimate country-specific effects for each NPI as most countries implemented most of their NPIs on the same day or in close succession.

Our study explains variation in the effectiveness of NPIs by estimating the association – and not the causal link – between the effectiveness of NPIs and country characteristics. However, it is still reasonable to compare the sign and magnitude of the observed associations with the expectation motivating the choice of the different country characteristics. The difference between association and causality is illustrated by similar associations for country characteristics that are highly correlated. For instance, a higher effectiveness of NPIs could be linked to a lower share of the young or to a higher share of the elderly population, or it could be linked to higher government effectiveness or income.

Finally, our sample of countries may underestimate variation in the effectiveness of NPIs. Although we analyze high-quality data from Brauner et al.^1^ covering a large sample of $n=40$ countries, the majority of the sample comprises European countries that pursued similar strategies in controlling the spread of COVID-19. Considering more countries pursuing a distinctive strategy such as Australia and Hong Kong may have induced more variation in the effectiveness of NPIs. In general, it cannot be ruled out that the sample of countries affects the estimated associations between NPI effectiveness and country characteristics to some extent.

### Concluding remarks

Altogether, we found that NPIs appeared to be overall more effective in some countries than in others. In particular, we found the wealth and demographic structure of a country can explain these differences. As such, our results can inform public health decision-makers about favorable or unfavorable conditions for curbing the spread of infectious diseases. Specifically, governments may be able to change some conditions (e. g. health expenditures) to create a more favorable environment for the effectiveness of NPIs by improving epidemic preparedness. Meanwhile, a better understanding of non-modifiable conditions (e. g. socioeconomic factors) is still relevant as our findings suggest that decision-makers need to assume a higher (or lower) NPI effectiveness when transferring results across countries. As such, our findings help governments when tailoring the choice of NPIs to the conditions they encounter in their respective country.

To translate our findings into health policy decisions, it would be helpful to also have knowledge about the varying effectiveness of specific, individual NPIs. The sample size of our study was too limited to address this question. Consequently, this should be a topic for future research involving even more countries or making use of regional differences within countries. Similarly, detailed insights from other fields about factors moderating the effects of interventions (e. g. mobility, adherence, individual susceptibility) could assist in understanding the causal links between country characteristics and NPI effectiveness.

## Supplementary Information

Supplementary Information.

## Data Availability

We collected data from publicly available data sources (Johns Hopkins Coronavirus Resource Center for epidemiological data; the study by Brauner et al.^1^ for policy measures; various sources for country characteristics as listed in Table 1). Data files together with reproducible code are available from https://github.com/nbanho/npi_effectiveness_predictors.
