# Supplementary material for: Estimating and explaining cross-country variation in the effectiveness of non-pharmaceutical interventions during COVID-19
Source: Sci Rep. 2022 May 9;12:7526. doi: 10.1038/s41598-022-11362-x (PMC9085796; doi:10.1038/s41598-022-11362-x)
Supplement: Supplementary file 1 — Supplementary Information. [file 41598_2022_11362_MOESM1_ESM.pdf]

Supplementary Information:
Estimating and explaining cross-country variation in the effectiveness of non-pharmaceutical interventions during COVID-19

Nicolas Banholzer[1*], Stefan Feuerriegel[1,2], and Werner Vach[3,4]

[1]ETH Zurich, Zurich, Switzerland

[2]LMU Munich, Munich, Germany

[3]Basel Academy for Quality and Research in Medicine, Basel, Switzerland

[4]University of Basel, Basel, Switzerland

[*]Corresponding author: nbanholzer@ethz.ch

Appendix

A Detailed prior and modeling choices 3

B Simulation-based study 5

C Descriptives 8

D Sensitivity analysis 9

Supplementary Figures

S1 Proportion of simulations containing the true country-specific NPI effect 6

S2 Estimated posterior means from simulation-based study 7

S3 Bivariate correlation (Pearson's r) between country-specific predictors. 8

S4 Sensitivity analysis: leave-one country-out analysis 9

Supplementary Tables

S1 List of priors for estimating the population-level effects of NPIs. 3

S2 List of priors for estimating the country-specific effects of NPIs and their univariate association with country-specific predictors. 4

S3 List of priors for the latent factor model used for joint estimation of the association between country-specific NPI effects and predictors. 4

A Detailed prior and modeling choices

In the following, we describe detailed prior and modeling choices.

Estimation is divided into a non-modeling phase and a modeling phase. In the non-modeling phase, the number of new cases are not modeled but the latent components are still computed. This takes into account high variability in the early number of cases as some countries still had to set up their documentation practices. As a result, the modeling phase starts after 100 cumulative cases were documented or seven days after the implementation of the first NPI, whatever comes first. The country-specific start of seven days after the first NPI is a small modification to accommodate the data by Brauner et al.[?], which consist of a number of small countries (e.g. Malta, Albania, Georgia) that otherwise reached 100 cumulative cases after most NPIs were already implemented.

Choices of priors for model parameters follow related work and general recommendations in Bayesian modeling[?]. Model parameters related to the original model for estimating the cross-country average effects of NPIs were given the priors listed in Table S1. Compared to the model in Banholzer et al.[?], the following modifications were made. For the effects of NPIs θ_m, the asymmetric Laplace prior from Brauner et al.[?] was chosen. To obtain better convergence of the model parameters on the data from Brauner et al., it was further necessary to place more informative priors on the over-dispersion parameter for the number of new infections and for the parameters concerning the time from infection to reporting of a new case. The former was informed by the posterior distribution from our original study, while the latter were informed by the less diffuse priors from Brauner et al.

Parameter	Prior
Country-specific transmission rate	$\alpha_j \sim \text{Normal}(\mu = \alpha, \sigma = \tau_1)$
	$\alpha \sim \text{Student-}t(\nu = 7, \mu = 0, \sigma = 10)$
	$\tau_1 \sim \text{Student-}t(\nu = 4, \mu = 0, \sigma = 1)$
Population-level NPI effects	$\theta_m \sim \text{Asym.} - \text{Laplace}(\mu = 0, \sigma = 10, \kappa = 0.5)$
Over-dispersion for the number of new infections	$\phi^I \sim \text{Normal}(\mu = 8.7, \sigma = 1)$
Over-dispersion for the number of new cases	$\frac{1}{\sqrt{\phi^N}} \sim \text{Normal}^+(\mu = 0, \sigma = 1)$
Number of new infections at epidemic start	$I_{jt_0} \sim \text{Exponential}(1/\lambda)$
	$\lambda \sim \text{Exponential}(1)$
Time from infection to new case	$p_{IN} \sim \text{Lognormal}(\mu_{p_{IN}}, \sigma_{p_{IN}})$
	$\mu_{p_{IN}} \sim \text{Normal}(10.92, 0.94)$
	$\sigma_{p_{IN}} \sim \text{Normal}(5.41, 0.27)$

Table S1. List of priors for estimating the population-level effects of NPIs.

Parameters linked to the country-specific effects θ_j and their univariate associations with country-specific predictors were given the priors listed in Table S2. Parameters involving the latent factor model for estimating the joint association between country-specific NPI effects and predictors were given the priors listed in Table S3.

Parameter	Prior
Country-specific NPI effect	$\theta_j \sim \text{Normal}(\mu = 0, \sigma = \tau_2)$
	$\tau_2 \sim \text{Student-}t(\nu = 4, \mu = 0, \sigma = 0.1)$
Association between α_j and θ_j	$\psi \sim \text{Student-}t(4, 0, 0.625)$
Association between θ_j and X_K	$\beta_k \sim \text{Student-}t(4, 0, 0.125)$
Residual variation in θ_j	$\epsilon_j \sim \text{Normal}(0, \tau_2)$
Missing predictor values	$\text{o} \sim \text{Normal}(0, 1)$

Table S2. List of priors for estimating the country-specific effects of NPIs and their univariate association with country-specific predictors.

Parameter	Prior
Latent factors	$Z \sim \text{Normal}(0, I_d)$
Weights on latent factors	$W_d \sim \text{Normal}(0, \omega_d)$
	$1/\sqrt{\omega_d} \sim \text{Gamma}(0.001, 0.001)$
Residual variation in latent factors	$\epsilon_k \sim \text{Normal}(0, \tau_3)$
	$\tau_3 \sim \text{Student-}t(4, 0, 1)$

Table S3. List of priors for the latent factor model used for joint estimation of the association between country-specific NPI effects and predictors.

B Simulation-based study

We conduct a simulation-based study in order to show that our model can recover the true country-specific NPI effects θ_j. The simulation-based study is conducted as follows. First, we set our model parameters to fixed values. For θ_j, we consider the scenario as described below. All other parameters are set to their posterior mean from the model without country-specific NPI effects. Second, we simulate data for the number of new cases from our model based on the NPIs that were implemented in each country over time. Third, the simulated number of new cases are used as model input for estimating the country-specific NPI effects.

The following effects are assumed for simulating data for the true country-specific NPI effects θ_j. In the probabilistic model, the θ_j are estimated hierarchically via partial pooling. This will shrink the country-specific effects towards their population mean when there is only small variation between countries, i.e. the data containing little information for strong country-specific NPI effects. Naturally, we would a priori expect less shrinkage for countries with more observed cases. Thus, in order to investigate the potentially asymmetric effect of shrinkage, we construct the following scenario. We first sort countries by their cumulative number of cases over the study period. Then, we assign large θ_j for both the countries with the smallest and largest cumulative number of cases. Further, we assign an equal share of positive and negative effects evenly across countries sorted by the cumulative number of cases. Thereby, a scenario is constructed that allows us to check the influence of the cumulative number of new cases and the sign of the effect on the amount of shrinkage.

A total of fifty simulated datasets is generated. The model parameters did not converge for one simulation, which was excluded. Thus, the results from the simulation-based study are based on the remaining 49 simulations. For the majority of countries, the true θ_j is recovered within the uncertainty implied by the fitted posterior distribution of our model (Fig. S1). Yet, there are some countries (e.g. Malta) where the estimated effects are often smaller than the true effect (Fig. S2). In comparison, the shrinkage seems larger for countries with low cumulative number of cases and for positive effect sizes. The former was expected while the latter may be attributed to the asymmetric prior for the cross-country NPI effects θ_m (see Supplement A).

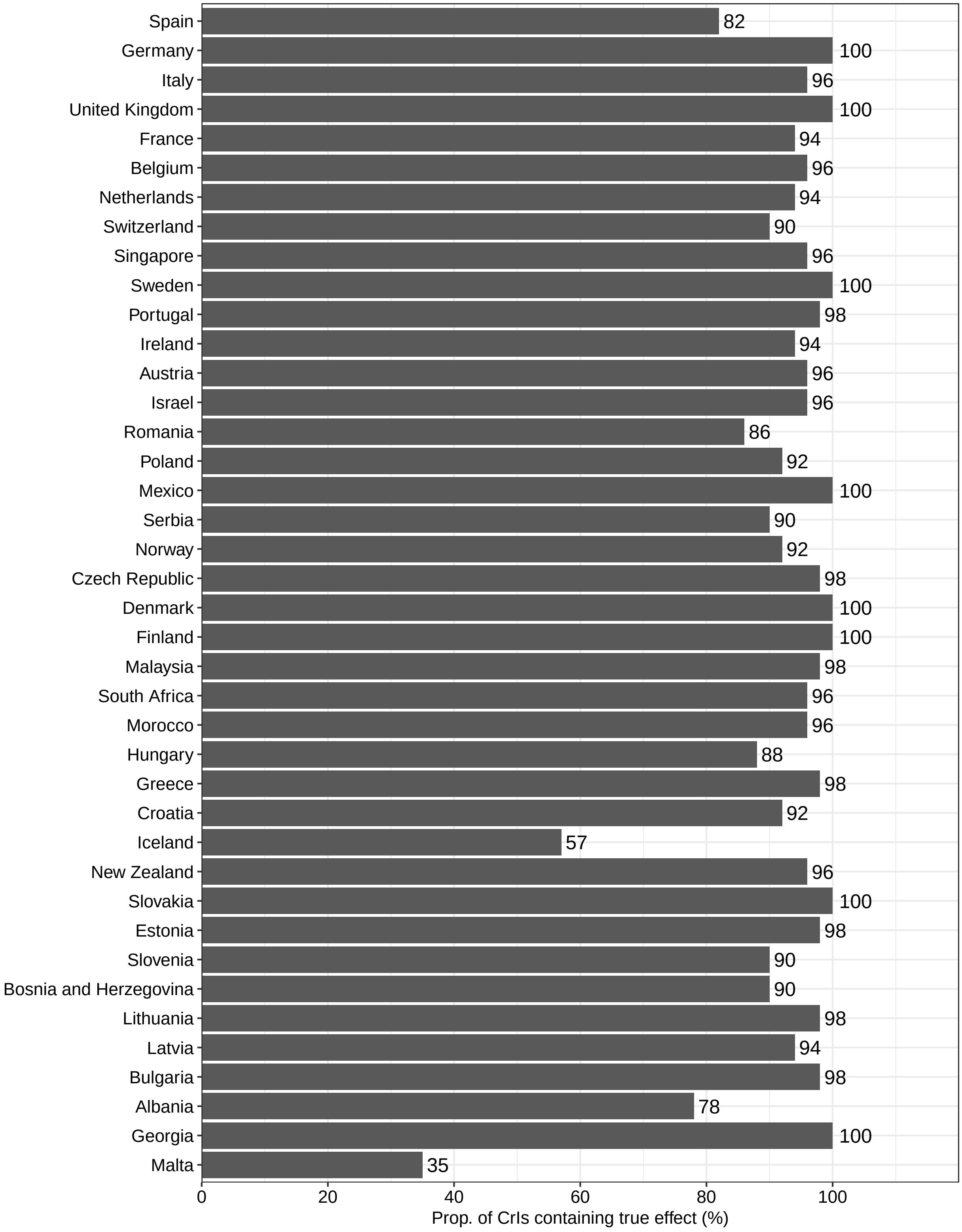

Fig S1. Proportion of simulations where the posterior credible intervals (CrIs) contain the true country-specific NPI effect.

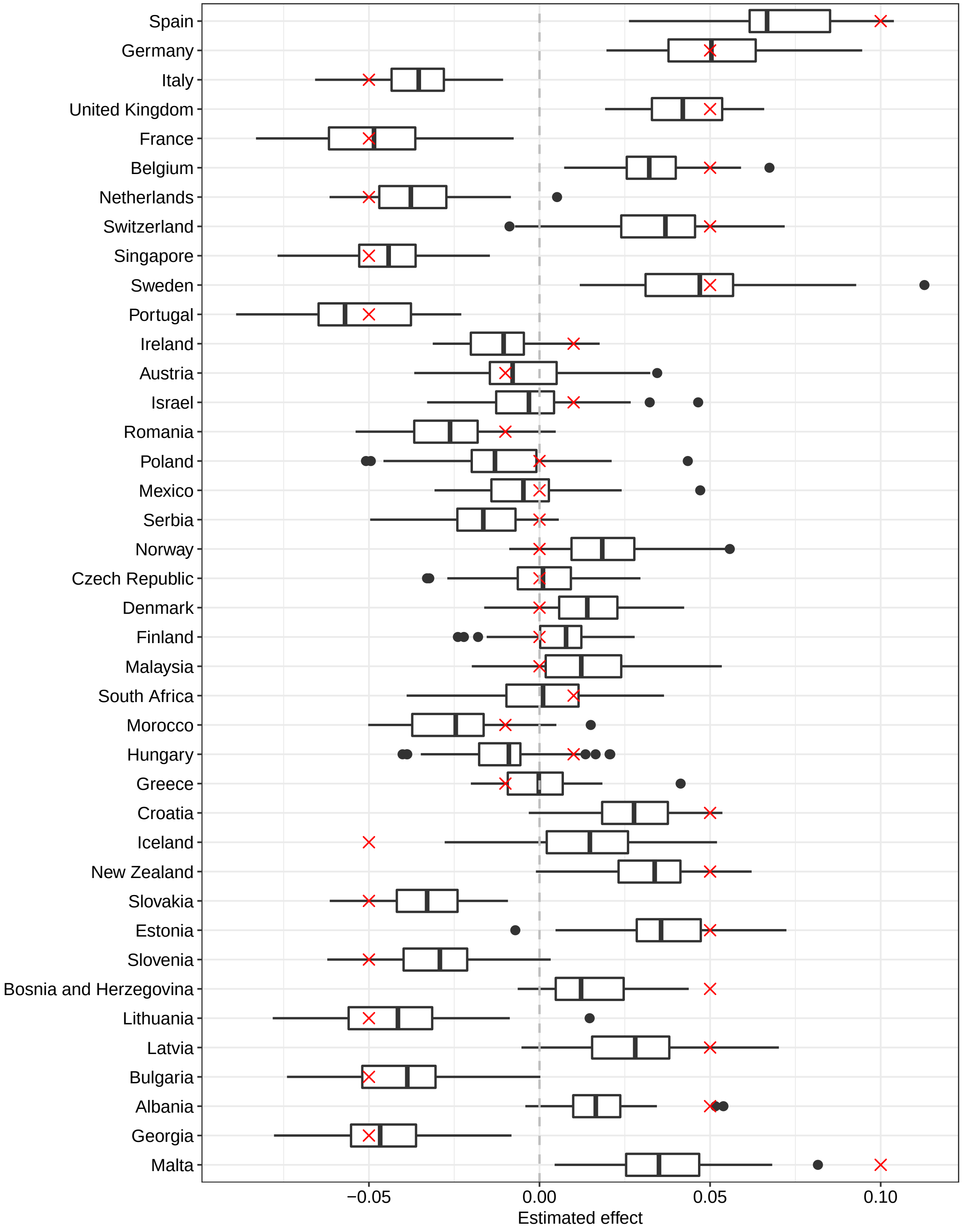

Fig S2. Estimated country-specific NPI effects from simulations (posterior means as boxplots and assumed true effect as red cross).

C Descriptives

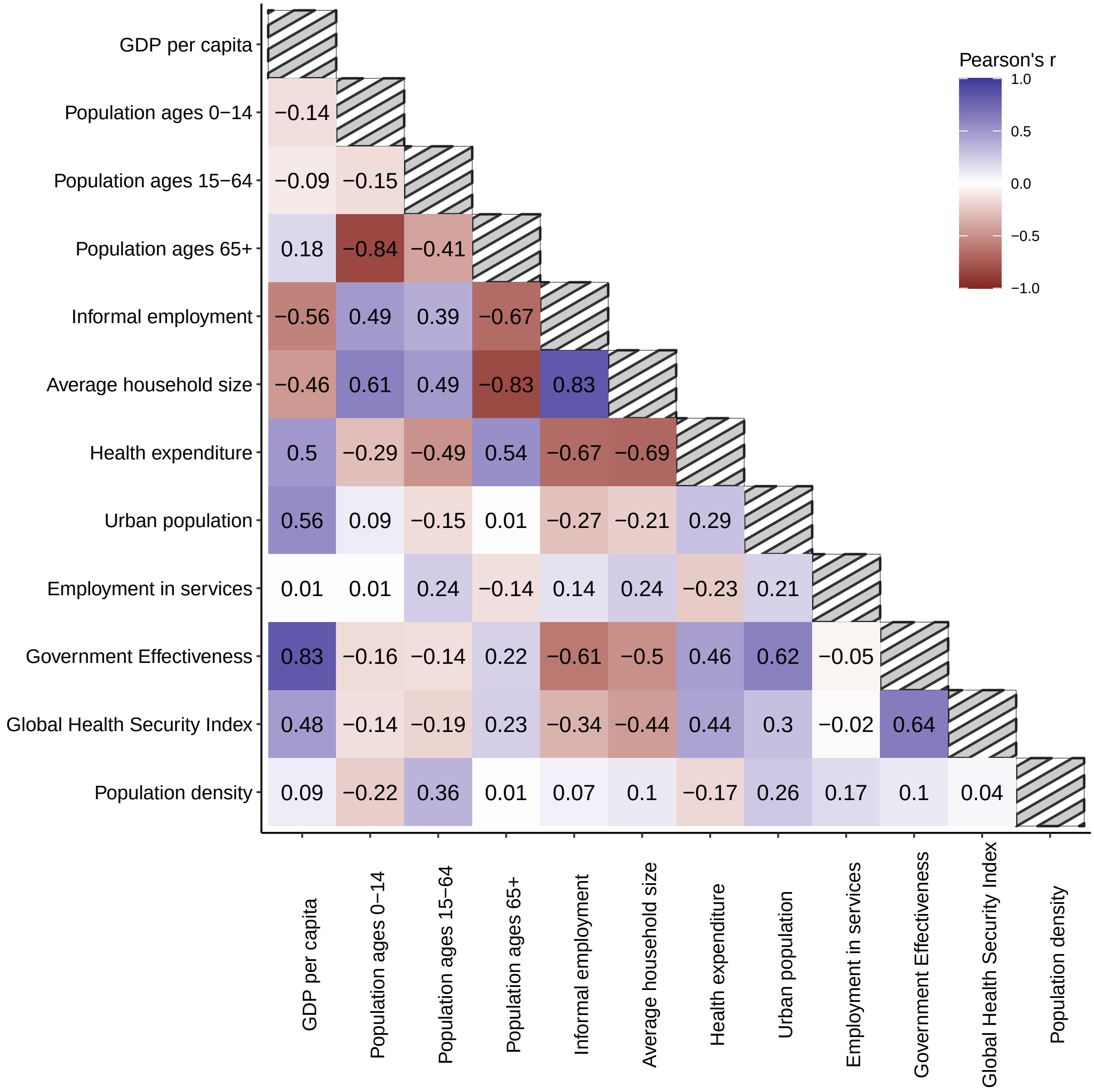

Fig S3. Bivariate correlation (Pearson's r) between country-specific predictors.

D Sensitivity analysis

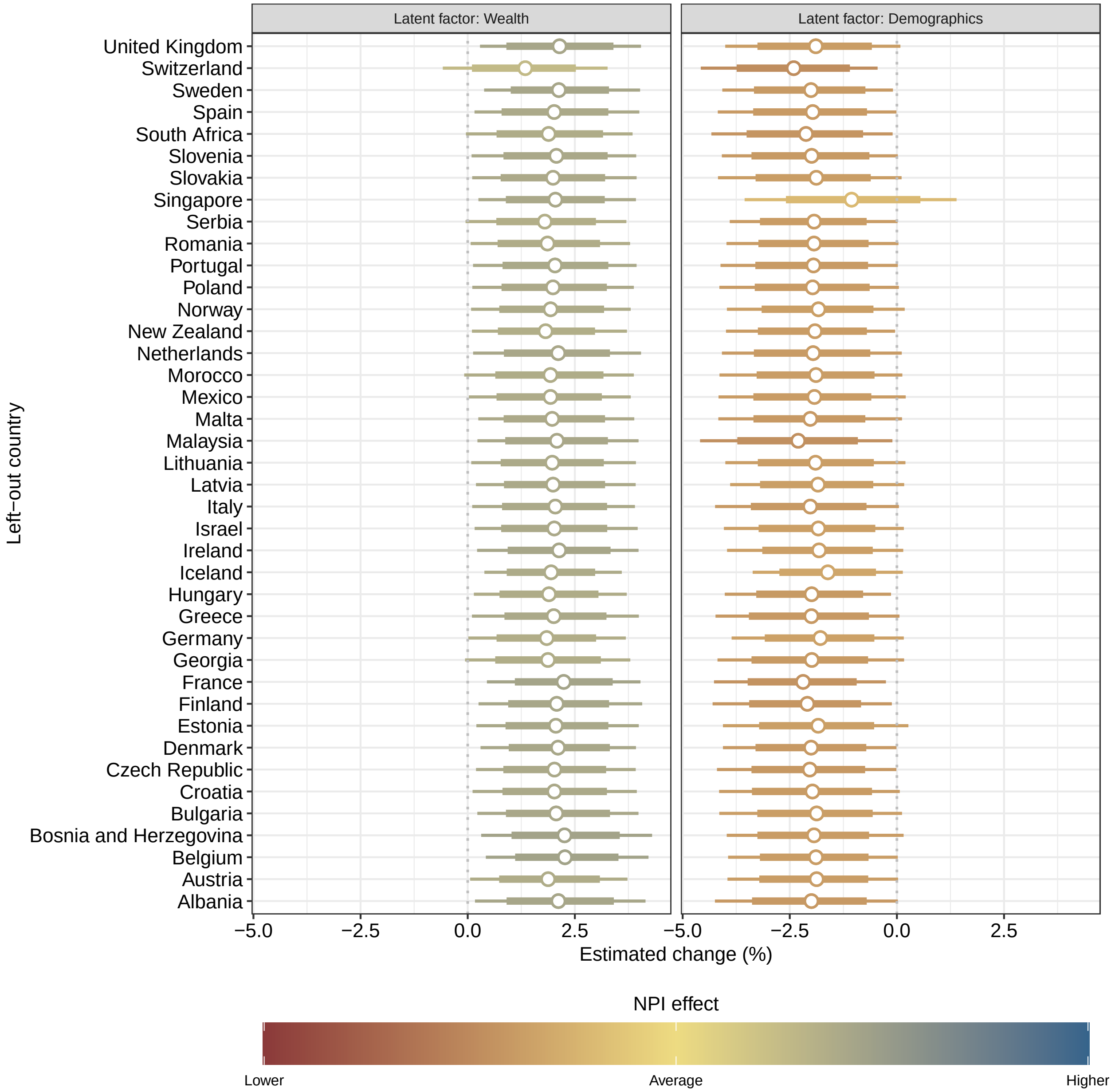

Fig S4. Sensitivity analysis: Estimated relative change (in %) in avoided new infections compared to the cross-country average effect of the single NPIs (posterior distribution with mean as dots and with 80% and 95% credible interval as thick and thin lines, respectively) for a +1 standard deviation (SD) increase in the latent factor when re-estimating the model leaving out one country at a time.
